# Puerperal Insanity

**Published:** 1909-11-27

**Authors:** 


### Mental Diseases.

### PUERPERAL INSANITY.

Of all attacks of mental disease that occur, those are fraught with possibly the most danger to the patient which commence during the puerperal period or soon after it. The special dangers lie in the facts that the onset is, as a rule, insidious, and that among the earliest symptoms are a growing dislike for those for whom most affection should be felt and a suicidal tendency. These early symptoms are generally carefully concealed by the patient, and often the first indication of anything wrong is some marked attack on a relative, or a determined attempt at suicide. Of all those that harm may be done to, the one that runs the gravest risk is, naturally, the baby. Reports of trials for murder of the child, committed under these circumstances, are not uncommon, but the cases in which the catastrophe was barely averted would, were they all to be reported, surprise and shock most people. The medical man is, of course, frequently blamed for not having warned the friends of the impending danger; but as a rule he is not only in no way to blame, but is less likely to notice small mental changes in his patient than the near relatives who are with her, and who know her better than he does. Besides this, one is always naturally loth to attribute small mental changes in an individual to anything else than normal variation, or to mention the fact if one does, since to the lay mind insanity is not a disease but an unfortunate stigma. This being so in ordinary circumstances, one is still more careful when observing anyone who is undergoing a normal reaction after a period of abnormal mental stress, as after a confinement. The blame, therefore, for not having called attention to minor mental changes which were not noticed by the friends, cannot be with justice thrown on the doctor.

Puerperal insanity is commoner after the first than after subsequent confinements; and is said to occur frequently after illegitimate pregnancies. The reason for this appears to be that the mental stress is greater during the first pregnancy than during later ones. The normal symptoms of pregnancy, besides being wearing, are new to the patient; there is an emotional state which is very strong, and which lasts for some months, becoming stronger as confinement approaches; and finally a period of much physical pain followed by a change of emotional state. Of this emotional state, the principal components appear to be fear, expectation, and joy; and of these, the two former disappear on delivery. Thus the normal mental stress is great, and it is sometimes further increased by croakings of ignorant friends, or the reading of books of the "Advice to Young Mothers" type which abound nowadays. In many cases is found an extra source of trouble in the pressure of family or financial worries. In illegitimate pregnancies there is, of course, a further emotional burden. The factors just enumerated can generally be found sufficient to ascribe as a cause; but in many cases none of them seem to have been of sufficient strength to have had anything to do with the breakdown, and in these one is often quite unable to account for it at all. Sometimes shock at the child being born dead or physically abnormal is the determining cause. Cases, it may be noted, following puerperal sepsis are not classed as puerperal, but as toxic insanity.

The symptoms may take the form of either mania or melancholia, but in the excited cases there is generally a very marked reactionary depression. Whichever form they take, the dislike for friends and relatives appears, and a suicidal impulse, except in the mildest cases. Some very mild cases display neither excitement nor depression, but simply appear to be of the confusional type. Hallucinations and delusions of all kinds occur, also insomnia and refusal of food. Constipation, as would be expected, is present, and often dirty tongue and foul breath. In the depression following maniacal symptoms, the suicidal tendency is very dangerous, and must be very carefully guarded against.

The majority of cases make good recoveries, and the greater number do not have any recurrence at subsequent confinements. Some few do not recover, but drift into a chronic state and finally into dementia. In these will usually be found a history of former attacks or a bad family history. Many of the patients who finally recover hang fire at some period of the illness, and remain without sign of improvement for a long time. The liability to this renders any prophecy as to the probable length of the attack both difficult and dangerous.

The indications for prevention are plain. As much freedom from all sources of worry as possible, attention to the general health, occupation enough to interest and to prevent too much thought being given to her own condition and feelings, and advice and help, when needed, from a sensible woman whom she can trust. Books of advice should not be allowed, as not only are they useless if there is someone to appeal to in case of need, but one so often hears from patients and friends of the baneful influence they have had on those who have studied them. The engagement of a capable nurse, and the knowledge that she will be at hand when required, will remove a good deal of anxiety at a time when it is least desirable.

At the first sign of the presence of mental symptoms the baby should be removed, and should not be left with the mother again until after complete recovery. It is generally best not to allow her to see it at all, but in mild cases, if she is anxious to do so, she may be allowed to, though she should not be permitted to nurse it. She should be left alone as little as possible on account of the frequent tendency to suicidal impulse, and all poisonous substances and objects with which she could do herself bodily harm should be removed. If food is refused, forcible feeding may become necessary, and should not be delayed. In the case of there being much secretion of milk, steps must be taken at once to stop it, and a close watch be kept for signs of mammary inflammation. Lochia cease with the onset of the mental symptoms, but nothing need be done for this unless there is some indication for a douche, which very rarely happens. At first patients are best kept in bed, unless there is any contra-indication; after a week or so they should be got up and given exercise in the open air. The case will be best nursed by those who have had training in nursing mental patients and who are strangers to her. If possible to manage at home, and if progress is made, certification is, of course, best avoided; but if improvement does not appear, a change from home, either in an institution or not, ought to be tried. It is best to insist on a long period of convalescence and change of air before allowing return to home duties.

